# Supplementary material for: Three-Dimensional Structural Heteromorphs of Mating-Type Proteins in Hirsutella sinensis and the Natural Cordyceps sinensis Insect–Fungal Complex
Source: J Fungi (Basel). 2025 Mar 23;11(4):244. doi: 10.3390/jof11040244 (PMC12028455; doi:10.3390/jof11040244)
Supplement: Supplementary file 1 [file jof-11-00244-s001.zip › jof-3449778-supplementary.pdf]

Supplement:

Three-Dimensional Structural Heteromorphs of Mating-Type Proteins in *Hirsutella sinensis* and the Natural *Cordyceps sinensis* Insect–Fungal Complex

Xiu-Zhang Li, Yu-Ling Li and Jia-Shi Zhu *

State Key Laboratory of Plateau Ecology and Agriculture, Qinghai Academy of Animal and Veterinary Sciences, Qinghai University, Xining 810016, China; xiuzhang11@163.com (X.-Z.L.); yulingli2000@163.com (Y.-L.L.)

Table S1. Amino acid scales based on the general chemical characteristics of their side chains for ProtScale analysis (https://web.expasy.org/protscale/) to predict secondary structures (α-helices, β-sheets, β-turns, and coils) of proteins.

			**Chemical-physical property**	**α-Helix**	**β-Sheet**	**β-Turn**	**Coil**
Aspartic acid	Asp	D	Acidic	0.924	0.541	1.197	1.197
Glutamic acid	Glu	E	Acidic	1.504	0.567	1.149	0.761
Alanine	Ala	A	Aliphatic	1.489	0.709	0.788	0.824
Isoleucine	Ile	I	Aliphatic	1.003	1.799	0.240	0.886
Leucine	Leu	L	Aliphatic	1.236	1.261	0.670	0.810
Valine	Val	V	Aliphatic	0.990	1.965	0.387	0.772
Phenylalanine	Phe	F	Aromatic	1.195	1.393	0.624	0.797
Tryptophan	Trp	W	Aromatic	1.090	1.306	0.546	0.941
Tyrosine	Tyr	Y	Aromatic	0.787	1.266	0.795	1.109
Arginine	Arg	R	Basic	1.224	0.920	0.912	0.893
Histidine	His	H	Basic	1.003	0.863	0.970	1.068
Lysine	Lys	K	Basic	1.172	0.721	1.302	0.897
Asparagine	Asn	N	with polar neutral side chains	0.772	0.604	1.572	1.167
Cysteine	Cys	C	with polar neutral side chains	0.966	1.191	0.965	0.953
Glutamine	Gln	Q	with polar neutral side chains	1.164	0.840	0.997	0.947
Methionine	Met	M	with polar neutral side chains	1.363	1.210	0.436	0.810
Serine	Ser	S	with polar neutral side chains	0.739	0.928	1.316	1.130

Threonine	Thr	T	with polar neutral side chains	0.785	1.221	0.739	1.148
Glycine	Gly	G	Unique amino acids	0.510	0.657	1.860	1.251
Proline	Pro	P	Unique amino acids	0.492	0.354	1.415	1.540

Note: An **amino acid scale** is defined at https://web.expasy.org/protscale/ by a numerical value assigned to each type of amino acid. The most frequently used scales are the hydrophobicity or hydrophilicity scales and the secondary structure conformational parameter scales, but many other scales exist, which are based on the different chemical and physical properties of the amino acids. The ExPASy ProtScale program provides 57 predefined scales based on the literature [Deleage, & Roux 1987].

```
AEH27625   1     MANPINMIPNPQWNATDYEAIWKGLEAQVNPFSQILCLEGDFFRQLDDAAKLFIARKLME   60
ACV60363   1     ------------------------------------------------------------   60
AFX66471   1     ------------------------------------------------------------   60
AFX66481   1     ------------------------------------------------------------   60
AFX66483   1     ------------------------------------------------------------   60
AFX66485   1     ------------------------------------------------------------   60
AFX66486   1     ------------------------------------------------------------   60

AEH27625   61    HVQESVLYVNDGNGPDRVYLGAPRHFVVGGGMILQISGYAPYWIRRSVSKVVTATVLAPP   120
ACV60363   61    ------------------------------------------------------------   120
AFX66471   61    ------------------------------------------------------------   120
AFX66481   61    ------------------------------------------------------------   120
AFX66483   61    ------------------------------------------------------------   120
AFX66485   61    ------------------------------------------------------------   120
AFX66486   61    ------------------------------------------------------------   120

AEH27625   121   SPKDIKIPRPPNAYILYRKERHHYVKDANPGITNNEISQILGKAWNMESNDVRQKYKDMS   180
ACV60363   121   -----------------------H------------------------------------   180
AFX66471   121   -----------------------H------------------------------------   180
AFX66481   121   -----------------------H------------------------------------   180
AFX66483   121   -----------------------H------------------------------------   180
AFX66485   121   -----------------------H------------------------------------   180
AFX66486   121   -----------------------H------------------------------------   180

AEH27625   181   QQVKQALLEKHPDYQYKPRRPCERRRRRRASPNQNPKQSTSRNAATRDAAISSEDTSTAT   240
ACV60363   181   ------------------------------------------------------------   240
AFX66471   181   ------------------------------------------------------------   240
AFX66481   181   ------------------------------------------------------------   240
AFX66483   181   ------------------------------------------------------------   240
AFX66485   181   ------------------------------------------------------------   240
AFX66486   181   ------------------------------------------------------------   240

AEH27625   241   GDTNTANGF                                                      249
ACV60363   241   ---------                                                      249
AFX66471   241   ---------                                                      249
AFX66481   241   ---------                                                      249
AFX66483   241   ---------                                                      249
AFX66485   241   ---------                                                      249
AFX66486   241   ---------                                                      249
```

Figure S1. Alignment of the full-length sequences of reference MAT1-2-1 proteins AEH27625 of structural morph Branch I-1 of Bayesian Cluster I, ACV60363 of structural morph Branch V-1 of Bayesian Cluster V, and 5 MAT1-2-1 proteins, namely, AFX66471, AFX66481, AFX66483, AFX66485, and AFX66486, for which there are no 3D structure records in the AlphaFold database. An HMG-

box_ROX1-like domain is highlighted in blue and underlined in the query protein sequence AEH27625 (127→197). The residues shown in green refer to conservative amino acid substitutions within the HMG-box_ROX1-like domain. The hyphens indicate identical amino acid residues.

[REFERENCES]

1. Abramson, J.; Adler, J.; Dunger, J.; Evans, R.; Green, T.; Pritzel, A.; Ronneberger, O.; Willmore, L.; Ballard, A.J.; Bambrick, J.; et al. Accurate structure prediction of biomolecular interactions with AlphaFold 3. *Nature* **2024**, *630*, 493–500. https://doi.org/10.1038/s41586-024-07487-w.
2. Barseghyan, G.S.; Holliday, J.C.; Price, T.C.; Madison, L.M.; Wasser, S.P. Growth and cultural-morphological characteristics of vegetative mycelia of medicinal caterpillar fungus *Ophiocordyceps sinensis* G.H. Sung et al. (Ascomycetes) Isolates from Tibetan Plateau (P. R. China). *Int. J. Med. Mushrooms* **2011**, *13*, 565–581. https://doi.org/10.1615/intjmedmushr.v13.i6.90.
3. Bennett, R.J.; Johnson, A.D. Completion of a parasexual cycle in *Candida albicans* by induced chromosome loss in tetraploid strains. *EMBO J.* **2003**, *22*, 2505–2515. https://doi.org/10.1093/emboj/cdg235.
4. Bushley, K.E.; Li, Y.; Wang, W.-J.; Wang, X.-L.; Jiao, L.; Spatafora, J.W.; Yao, Y.-J. Isolation of the MAT1-1 mating type idiomorph and evidence for selfing in the Chinese medicinal fungus *Ophiocordyceps sinensis*. *Fungal Biol.* **2013**, *117*, 599–610. https://doi.org/10.1016/j.funbio.2013.06.001.
5. Chen, C.-S.; Hseu, R.-S.; Huang, C.-T. Quality control of *Cordyceps sinensis* teleomorph, anamorph, and Its products. In *Quality Control of Herbal Medicines and Related Areas*; Shoyama, Y., Ed.; InTech: Rijeka, Croatia, 2011; Chapter 12, pp. 223–238. Available online: www.intechopen.com (accessed on 30 January 2025).
6. Chen, Y.-Q.; Hu, B.; Xu, F.; Zhang, W.; Zhou, H.; Qu, L.-H. Genetic variation of *Cordyceps sinensis*, a fruit-body-producing entomopathogenic species from different geographical regions in China. *FEMS Microbiol Lett.* **2004**, *230*, 153–158. https://doi.org/10.1016/S0378-1097(03)00889-9.
7. China Ministry of Agriculture and Rural Affairs. Announcement (No. 15 of 2021) of National Forestry and Grassland Administration: List of National Key Protected Wild Plants. 7 September 2021. Available online: https://www.forestry.gov.cn/c/www/gkml/11057.jhtml (accessed on 30 January 2025).
8. Dai, R.-Q.; Lan, J.-L.; Chen, W.-H.; Li, X.-M.; Chen, Q.-T.; Shen, C.-Y. Discovery of a new fungus *Paecilomyces hepiali* Chen & Dai. *Acta Agricult. Univ. Pekin.* **1989**, *15*, 221–224.
9. David, A.; Islam, S.; Tankhilevich, E.; Sternberg, M.J.E. The AlphaFold Database of Protein Structures: A Biologist's Guide. *J. Mol. Biol.* **2022**, *434*, 167336. https://doi.org/10.1016/j.jmb.2021.167336.
10. Debuchy, R.; Turgeo, B.G. Mating-Type Structure, Evolution, and Function in Euascomycetes. In *Growth, Differentiation and Sexuality*; Kües, U., Fischer, R., Eds.; Springer: Berlin/Heidelberg, Germany, 2006; pp. 293–323.
11. Deleage, G.; Roux, B. An algorithm for protein secondary structure prediction based on class prediction. *Protein Eng. Des. Sel.* **1987**, *1*, 289–294. https://doi.org/10.1093/protein/1.4.289.
12. Du, X.-H.; Wu, D.-M.; Kang, H.; Wang, H.-C.; Xu, N.; Li, T.-T.; Chen, K.-L. Heterothallism and potential hybridization events inferred for twenty-two yellow morel species. *IMA Fungus* **2020**, *11*, 4. https://doi.org/10.1186/s43008-020-0027-1.
13. Engh, I.B. Molecular Phylogeny of the *Cordyceps-Tolypocladium* Complex. PhD. Thesis, Department of Biology, University of Oslo, Oslo, Norway, 1999.
14. Gasteiger, E.; Hoogland, C.; Gattiker, A.; Duvaud, S.; Wilkins, M.R.; Appel, R.D.; Bairoch, A. Protein Identification and Analysis Tools on the ExPASy Server. In *The Proteomics Protocols Handbook*; Walker, J.M., ed.; Humana Press: Totowa, NJ, USA, 2005; Chapter 52, pp. 571–607.
15. Guo, M.-Y.; Liu, Y.; Gao, Y.-H.; Jin, T.; Zhang, H.-B.; Zhou, X.-W. Identification and bioactive potential of endogenetic fungi isolated from medicinal caterpillar fungus *Ophiocordyceps sinensis* from Tibetan Plateau. *Int. J. Agric. Biol.* **2017**, *19*, 307–313. https://doi.org/10.17957/IJAB/15.0281.

16. Hawksworth, D.L.; Crous, P.W.; Redhead, S.A.; Reynolds, D.R.; Samson, R.A.; Seifert, K.A.; Taylor, J.W.; Wingfield, M.J.; Abaci, Ö.; Aime, C.; et al. The Amsterdam declaration on fungal nomenclature. *IMA Fungus* **2011**, *2*, 105–112. https://doi.org/10.5598/imafungus.2011.02.01.14.
17. Hėnault, M.; Marsit, S.; Charron, G.; Landry, C.R. The effect of hybridization on transposable element accumulation in an undomesticated fungal species. *eLife* **2020**, *9*, e60474. https://doi.org/10.7554/eLife.60474.
18. Holliday, J.; Cleaver, M. Medicinal value of the caterpillar fungi species of the genus Cordyceps (Fr.) Link (Ascomycetes). A review. *Int. J. Med. Mushrooms* **2008**, *10*, 219–234. https://doi.org/10.1615/IntJMedMushr.v10.i3.30.
19. Hu, X.; Zhang, Y.-J.; Xiao, G.-H.; Zheng, P.; Xia, Y.-L.; Zhang, X.-Y.; St Leger, R.J.; Liu, X.-Z.; Wang, C.-S. Genome survey uncovers the secrets of sex and lifestyle in caterpillar fungus. *Chin. Sci. Bull.* **2013**, *58*, 2846–2854. https://doi.org/10.1007/s11434-013-5929-5.
20. Huelsenbeck, J.P.; Ronquist, F. MRBAYES: Bayesian inference of phylogeny. *Bioinformatics* **2001**, *17*, 754–755. https://doi.org/10.1093/bioinformatics/17.8.754.
21. Jacobsen, S.; Wittig, M.; Pöggeler, S. Interaction Between Mating-Type Proteins From the Homothallic Fungus Sordaria macrospora. *Curr. Genet.* **2002**, *41*, 150–158. https://doi.org/10.1007/s00294-002-0276-0.
22. Jiang, Y.; Yao, Y.-J. A review for the debating studies on the anamorph of *Cordyceps sinensis*. *Mycosistema* **2003**, *22*, 161–176.
23. Jin, L.-Q.; Xu, Z.-W.; Zhang, B.; Yi, M.; Weng, C.-Y.; Lin, S.; Wu, H.; Qin, X.-T.; Xu, F.; Teng, Y.; et al. Genome sequencing and analysis of fungus *Hirsutella sinensis* isolated from *Ophiocordyceps sinensis*. *AMB Expr.* **2020**, *10*, 105. https://doi.org/10.1186/s13568-020-01039-x.
24. Jones, S.K.; Bennett, R.J. Fungal mating pheromones: Choreographing the dating game. *Fungal Genet. Biol.* **2011**, *48*, 668–676. https://doi.org/10.1016/j.fgb.2011.04.001.
25. Jumper, J.; Evans, R.; Pritzel, A.; Green, T.; Figurnov, M.; Ronneberger, O.; Tunyasuvunakool, K.; Bates, R.; Žídek, A.; Potapenko, A.; et al. Highly accurate protein structure prediction with AlphaFold. *Nature* **2021**, *596*, 583–589. https://doi.org/10.1038/s41586-021-03819-2.
26. Kang, Q.; Zhang, J.; Chen, F.; Dong, C.; Qin, Q.; Li, X.; Wang, H.; Zhang, H.; Meng, Q. Unveiling mycoviral diversity in *Ophiocordyceps sinensis* through transcriptome analyses. *Front. Microbiol.* **2024**, *15*, 1493365. https://doi.org/10.3389/fmicb.2024.1493365.
27. Kinjo, N.; Zang, M. Morphological and phylogenetic studies on *Cordyceps sinensis* distributed in southwestern China. *Mycoscience* **2001**, *42*, 567–574. https://doi.org/10.1007/BF02460956.
28. Kück, U.; Bennett, R.J.; Wang, L.; Dyer, P.S. Editorial: Sexual and Parasexual Reproduction of Human Fungal Pathogens. *Front. Cell. Infect. Microbiol.* **2022**, *12*, 934267. https://doi.org/10.3389/fcimb.2022.934267.
29. Leung, P.-H.; Zhang, Q.-X.; Wu, J.-Y. Mycelium cultivation, chemical composition and antitumour activity of a *Tolypocladium* sp. fungus isolated from wild *Cordyceps sinensis*. *J. Appl. Microbiol.* **2006**, *101*, 275–283. https://doi.org/10.1111/j.1365-2672.2006.02930.x.
30. Li, C.-L. A study of *Tolypocladium sinense* C.L. Li. sp. nov. and cyclosporin production. *Acta Mycol. Sinica* **1988**, *7*, 93–98.
31. Li, M.-M.; Zhang, J.-H.; Qin, Q.-L.; Zhang, H.; Li, X.; Wang, H.-T.; Meng, Q. Transcriptome and Metabolome Analyses of Thitarodes xiaojinensis in Response to *Ophiocordyceps sinensis* Infection. *Microorganisms* **2023**, *11*, 2361. https://doi.org/10.3390/microorganisms11092361.
32. Li, X.; Wang, F.; Liu, Q.; Li, Q.-P.; Qian, Z.-M.; Zhang, X.-L.; Li, K.; Li, W.-J.; Dong, C.-H. Developmental transcriptomics of Chinese cordyceps reveals gene regulatory network and expression profiles of sexual development-related genes. *BMC Genom.* **2019**, *20*, 337. https://doi.org/10.1186/s12864-019-5708-z.
33. Li, X.-Z.; Li, Y.-L.; Wang, Y.-N.; Zhu, J.-S. Translations of mutant repetitive genomic sequences in *Hirsutella sinensis* and changes in secondary structures and functional specifications of the encoded proteins. *Int. J. Mol. Sci.* **2024**, *25*, 11178. https://doi.org/10.3390/ijms252011178.
34. Li, X.-Z.; Li, Y.-L.; Yao, Y.-S.; Xie, W.-D.; Zhu, J.-S. Further discussion with Li et al. (2013, 2019) regarding the "ITS pseudogene hypothesis" for *Ophiocordyceps sinensis*. *Mol. Phylogenet. Evol.* **2020**, *146*, 106728. https://doi.org/10.1016/j.ympev.2019.106728.

35. Li, X.-Z.; Li, Y.-L.; Zhu, J.-S. Differential transcription of mating-type genes during sexual reproduction of natural *Cordyceps sinensis*. *Chin. J. Chin. Mater. Medica* **2023**, *48*, 2829–2840. https://doi.org/10.19540/j.cnki.cjcmm.20230213.102.
36. Li, X.-Z.; Xiao, M.-J.; Li, Y.-L.; Gao, L.; Zhu, J.-S. Mutations and differential transcription of mating-type and pheromone receptor genes in *Hirsutella sinensis* and the natural *Cordyceps sinensis* insect–fungi complex. *Biology* **2024**, *13*, 632 https://doi.org/10.3390/biology13080632.
37. Li, Y.; Hsiang, T.; Yang, R.-H.; Hu, X.-D.; Wang, K.; Wang, W.-J.; Wang, X.-L.; Jiao, L.; Yao, Y.-J. Comparison of different sequencing and assembly strategies for a repeat-rich fungal genome, *Ophiocordyceps sinensis*. *J. Microbiol. Methods* **2016**, *128*, 1−6. https://doi.org/10.1016/j.mimet.2016.06.025.
38. Li, Y.; Jiao, L.; Yao, Y.-J. Non-concerted ITS evolution in fungi, as revealed from the important medicinal fungus *Ophiocordyceps sinensis*. *Mol. Phylogenet. Evol.* **2013**, *68*, 373–379. https://doi.org/10.1016/j.ympev.2013.04.010.
39. Li, Y.-L.; Gao, L.; Yao, Y.-S.; Wu, Z.-M.; Lou, Z.-Q.; Xie, W.-D.; Wu, J.-Y.; Zhu, J.-S. Altered GC- and AT-biased genotypes of *Ophiocordyceps sinensis* in the stromal fertile portions and ascospores of natural *Cordyceps sinensis*. *PLoS ONE* **2023**, *18*, e0286865. https://doi.org/10.1371/journal.pone.0286865.
40. Li, Y.-L.; Li, X.-Z.; Yao, Y.-S.; Wu, Z.-M.; Gao, L.; Tan, N.-Z.; Lou, Z.-Q.; Xie, W.-D.; Wu, J.-Y.; Zhu, J.-S. Differential cooccurrence of multiple genotypes of *Ophiocordyceps sinensis* in the stromata, stromal fertile portion (ascocarps) and ascospores of natural *Cordyceps sinensis*. *PLoS ONE* **2023**, *18*, e0270776. https://doi.org/10.1371/journal.pone.0270776.
41. Li, Y.-L.; Li, X.-Z.; Yao, Y.-S.; Xie, W.-D.; Zhu, J.-S. Molecular identification of *Ophiocordyceps sinensis* genotypes and the indiscriminate use of the Latin name for the multiple genotypes and the natural insect-fungi complex. *Am. J. BioMed Sci.* **2022**, *14*, 115–135. https://doi.org/10.5099/aj220300115.
42. Li, Y.-L.; Yao, Y.-S.; Zhang, Z.-H.; Xu, H.-F.; Liu, X.; Ma, S.-L.; Wu, Z.-M.; Zhu, J.-S. Synergy of fungal complexes isolated from the intestines of *Hepialus lagii* larvae in increasing infection potency. *J. Fungal Res.* **2016**, *14*, 96−112.
43. Liu, J.; Guo, L.-N.; Li, Z.-W.; Zhou, Z.; Li, Z.; Li, Q.; Bo, X.-C.; Wang, S.-Q.; Wang, J.-L.; Ma, S.-C.; et al. Genomic analyses reveal evolutionary and geologic context for the plateau fungus *Ophiocordyceps sinensis*. *Clin. Med.* **2020**, *15*, 107–119. https://doi.org/10.1186/s13020-020-00365-3.
44. Liu, Z.-Q.; Lin, S.; Baker, P.J.; Wu, L.-F.; Wang, X.-R.; Wu, H.; Xu, F.; Wang, H.-Y.; Brathwaite, M.E.; Zheng, Y.-G. Transcriptome sequencing and analysis of the entomopathogenic fungus *Hirsutella sinensis* isolated from *Ophiocordyceps sinensis*. *BMC Genom.* **2015**, *16*, 106–123. https://doi.org/10.1186/s12864-015-1269-y.
45. Lu, H.-L.; St Leger, R.J. *Chapter Seven—Insect Immunity to Entomopathogenic Fungi*; Lovett, B., St Leger, R.J., Eds.; Advanc Genet; Academic Press: Cambridge, MA, USA, 2016; Volume 94; pp. 251–285.
46. Mariani, V.; Biasini, M.; Barbato, A.; Schwede, T. lDDT: A local superposition-free score for comparing protein structures and models using distance difference tests. *Bioinformatics* **2013**, *29*, 2722–2728. https://doi.org/10.1093/bioinformatics/btt473.
47. Mao, X.-M.; Zhao, S.-M.; Cao, L.; Yan, X.; Han, R.-C. The morphology observation of *Ophiocordyceps sinensis* from different origins. *J. Environ. Entomol.* **2013**, *35*, 343–353.
48. Meng, Q.; Yu, H.-Y.; Zhang, H.; Zhu, W.; Wang, M.-L.; Zhang, J.-H.; Zhou, G.-L.; Li, X.; Qin, Q.-L.; Hu, S.-N.; et al. Transcriptomic insight into the immune defenses in the ghost moth, *Hepialus xiaojinensis*, during an *Ophiocordyceps sinensis* fungal infection. Insect Biochem. *Mol. Biol.* **2015**, *64*, 1−15. https://doi.org/10.1016/j.ibmb.2015.06.014.
49. Mishra, A.; Forche, A.; Anderson, M.Z. Parasexuality of Candida Species. *Front. Cell. Infect. Microbiol.* **2021**, *11*, 796929. https://doi.org/10.3389/fcimb.2021.796929.
50. Monzon, V.; Haft, D.H.; Bateman, A. Folding the unfoldable: Using AlphaFold to explore spurious proteins. *Bioinform. Adv.* **2022**, *1*, vbab043. https://doi.org/10.1093/bioadv/vbab043.
51. Nakamura, N.; Tanaka, C.; Takeuchi-Kaneko, Y. Transmission of antibiotic-resistance markers by hyphal fusion suggests partial presence of parasexuality in the root endophytic fungus Glutinomyces brunneus. *Mycol. Prog.* **2019**, *18*, 453–462. https://doi.org/10.1007/s11557-018-1455-9.
52. Pfennig, K.S. Facultative Mate Choice Drives Adaptive Hybridization. *Science* **2007**, *318*, 965–967. https://doi.org/10.1126/science.1146035.

53. Peters, C.; Elofsson, A. Why is the biological hydrophobicity scale more accurate than earlier experimental hydrophobicity scales? *Proteins* **2014**, *82*, 2190–2198. https://doi.org/10.1002/prot.24582.
54. Rams, B.; Kück, U. The *Penicillium chrysogenum tom1* gene a major target of transcription factor MAT1-1-1 encodes a nuclear protein involved in sporulation. *Front. Fungal Biol.* **2022**, *3*, 937023. https://doi.org/10.3389/ffunb.2022.937023.
55. Ren, Y.; Wan, D.-G.; Lu, X.-M.; Guo, J.-L. The study of scientific name discussion for TCM Cordyceps. *LisShenzhen Med. Mater. Medica Res.* **2013**, *24*, 2211–2212.
56. Rettie, S.A.; Campbell, K.V.; Bera, A.K.; Kang, A.; Kozlov, S.; De La Cruz, J.; Adebomi, V.; Zhou, G.; DiMaio, F.; Ovchinnikov, S.; et al. Cyclic peptide structure prediction and design using AlphaFold. *bioRxiv* 2023, *Preprint*. https://doi.org/10.1101/2023.02.25.529956.
57. Ronquist, F.; Teslenko, M.; van der Mark, P.; Ayres, D.L.; Darling, A.; Höhna, S.; Larget, B.; Liu, L.; Suchard, M.A.; Huelsenbeck, J.P. MrBayes 3.2: Efficient Bayesian Phylogenetic Inference and Model Choice Across a Large Model Space, Systematic Biology, **2012**, *61*, 539–542. https://doi.org/10.1093/sysbio/sys029.
58. Samarasinghe, H.; You, M.; Jenkinson, T.S.; Xu, J.-P.; James, T.Y. Hybridization Facilitates Adaptive Evolution in Two Major Fungal Pathogens. *Genes* **2020**, *11*, 101. https://doi.org/10.3390/genes11010101.
59. Seervai, R.N.H.; Jones, S.K.; Hirakawa, M.P.; Porman, A.M.; Bennett, R.J. Parasexuality and ploidy change in *Candida tropicalis*. *Eukaryot. Cell* **2013**, *12*, 1629–1640. https://doi.org/10.1128/EC.00128-13.
60. Sherwood, R.K.; Bennett, R.J. Fungal meiosis and parasexual reproduction--lessons from pathogenic yeast. *Curr. Opin. Microbiol.* **2009**, *12*, 599–607. https://doi.org/10.1016/j.mib.2009.09.005.
61. Shu, R.-H.; Zhang, J.-H.; Meng, Q.; Zhang, H.; Zhou, G.-L.; Li, M.-M.; Wu, P.-P.; Zhao, Y.-N.; Chen, C.; Qin, Q.-L. A new high-quality draft genome assembly of the Chinese cordyceps *Ophiocordyceps sinensis*. *Genome Biol. Evol.* **2020**, *12*, 1074–1079. https://doi.org/10.1093/gbe/evaa112.
62. Simm, S.; Einloft, J.; Mirus, O.; Schleiff, E. 50 years of amino acid hydrophobicity scales: Revisiting the capacity for peptide classification. *Biol. Res.* **2016**, *49*, 31. https://doi.org/10.1186/s40659-016-0092-5.
63. Steensels, J.; Gallone, B.; Verstrepen, K.J. Interspecific hybridization as a driver of fungal evolution and Adaptation. *Nat. Rev. Microbiol.* **2021**, *19*, 485–500.
64. Stensrud, Ø.; Hywel-Jones, N.L.; Schumacher, T. Towards a phylogenetic classification of Cordyceps: ITS nrDNA sequence data confirm divergent lineages and paraphyly. *Mycol. Res.* **2005**, *109*, 41–56. https://doi.org/10.1017/s095375620400139x.
65. Stensrud, Ø.; Schumacher, T.; Shalchian-Tabrizi, K.; Svegardenib, I.B.; Kauserud, H. Accelerated nrDNA evolution and profound AT bias in the medicinal fungus *Cordyceps sinensis*. *Mycol. Res.* **2007**, *111*, 409–415. https://doi.org/10.1016/j.mycres.2007.01.015.
66. Stone, R. Improbable partners aim to bring biotechnology to a Himalayan kingdom. *Science* **2010**, *327*, 940–941. https://doi.org/10.1126/science.327.5968.940.
67. Sung, G.-H.; Hywel-Jones, N.L.; Sung, J.-M.; Luangsa-ard, J.J.; Shrestha, B.; Spatafora, J.W. Phylogenetic classification of *Cordyceps* and the clavicipitaceous fungi. *Stud. Mycol.* **2007**, *57*, 5–59. https://doi.org/10.3114/sim.2007.57.01.
68. Tunyasuvunakool, K.; Adler, J.; Wu, Z.; Green, T.; Zielinski, M.; Žídek, A.; Bridgland, A.; Cowie, A.; Meyer, C.; Laydon, A.; et al. Highly accurate protein structure prediction for the human proteome. *Nature* **2021**, *596*, 590–596. https://doi.org/10.1038/s41586-021-03828-1.
69. Turgeon, B.G.; Yoder, O.C. Proposed nomenclature for mating type genes of filamentous ascomycetes. *Fungal Genet. Biol.* **2000**, *31*, 1–5. https://doi.org/10.1006/fgbi.2000.1227.
70. Varadi, M.; Bertoni, D.; Magana, P.; Paramval, U.; Pidruchna, I.; Radhakrishnan, M.; Tsenkov, M.; Nair, S.; Mirdita, M.; Yeo, J.; et al. AlphaFold Protein Structure Database in 2024: Providing structure coverage for over 214 million protein sequences. *Nucleic Acids Res.* **2024**, *52*, D368–D375. https://doi.org/10.1093/nar/gkad1011.
71. Wang, Y.; Stata, M.; Wang, W.; Stajich, J.E.; White, M.M.; Moncalvo, J.M. Comparative genomics reveals the core gene toolbox for the fungus-insect symbiosis. *mBio* **2018**, *9*, e00636-18. https://doi.org/10.1128/mBio.00636-18.

72. Wang, Y.-B.; Wang, Y.; Fan, Q.; Duan, D.-E.; Zhang, G.-D.; Dai, R.-Q.; Dai, Y.-D.; Zeng, W.-B.; Chen, Z.-H.; Li, D.-D.; et al. Multigene phylogeny of the family Cordycipitaceae (Hypocreales): New taxa and the new systematic position of the Chinese cordycipitoid fungus *Paecilomyces hepiali*. *Fungal Divers* **2020**, *103*, 1. https://doi.org/10.1007/s13225-020-00457-3.
73. Wei, J.-C.; Wei, X.-L.; Zheng, W.-F.; Guo, W.; Liu, R.-D. Species identification and component detection of *Ophiocordyceps sinensis* cultivated by modern industry. *Mycosystema* **2016**, *35*, 404–410.
74. Wei, X.-L.; Yin, X.-C.; Guo, Y.-L.; Shen, N.-Y.; Wei, J.-C. Analyses of molecular systematics on *Cordyceps sinensis* and its related taxa. *Mycosystema* **2006**, *25*, 192–202.
75. Wilson, A.M.; Wilken, P.M.; van der Nest, M.A.; Steenkamp, E.T.; Wingfield, M.J.; Wingfield, B.D. Homothallism: An umbrella term for describing diverse sexual behaviours. *IMA Fungus* **2015**, *6*, 207–214. https://doi.org/10.5598/imafungus.2015.06.01.13.
76. Wroblewski, K.; Kmiecik, S. Integrating AlphaFold pLDDT Scores into CABS-flex for enhanced protein flexibility simulations. *Comput. Struct. Biotechnol. J.* **2024**, *30*, 4350–4356. https://doi.org/10.1016/j.csbj.2024.11.047.
77. Xia, E.-H.; Yang, D.-R.; Jiang, J.-J.; Zhang, Q.-J.; Liu, Y.; Liu, Y.-L.; Zhang, Y.; Zhang, H.-B.; Shi, C.; Tong, Y.; et al. The caterpillar fungus, *Ophiocordyceps sinensis*, genome provides insights into highland adaptation of fungal pathogenicity. *Sci. Rep.* **2017**, *7*, 1806. https://doi.org/10.1038/s41598-017-01869-z.
78. Xia, F.; Liu, Y.; Shen, G.-L.; Guo, L.-X.; Zhou, X.-W. Investigation and analysis of microbiological communities in natural *Ophiocordyceps sinensis*. *Can. J. Microbiol.* **2015**, *61*, 104–111. https://doi.org/10.1139/cjm-2014-0610.
79. Xiang, L.; Li, Y.; Zhu, Y.; Luo, H.; Li, C.; Xu, X.; Sun, C.; Song, J.-Y.; Shi, L.-H.; He, L.; et al. Transcriptome analysis of the *Ophiocordyceps sinensis* fruiting body reveals putative genes involved in fruiting body development and cordycepin biosynthesis. *Genomics* **2014**, *103*, 154−159. https://doi.org/10.1016/j.ygeno.2014.01.002.
80. Xiao, W.; Yang, J.-P.; Zhu, P.; Cheng, K.-D.; He, H.-X.; Zhu, H.-X.; Wang, Q. Non-support of species complex hypothesis of *Cordyceps sinensis* by targeted rDNA-ITS sequence analysis. *Mycosystema* **2009**, *28*, 724–730.
81. Xu, T.; Xu, Q.; Li, J.-Y. Toward the appropriate interpretation of Alphafold2. *Front. Artif. Intell.* **2023**, *6*, 1149748. https://doi.org/10.3389/frai.2023.1149748.
82. Yang, J.-L.; Xiao, W.; He, H.-X.; Zhu, H.-X.; Wang, S.-F.; Cheng, K.-D.; Zhu, P. Molecular phylogenetic analysis of *Paecilomyces hepiali* and *Cordyceps sinensis*. *Acta Pharmaceut. Sinica* **2008**, *43*, 421–426.
83. Yang, J.-Y.; Tong, X.-X.; He, C.-Y.; Bai, J.; Wang, F.; Guo, J.-L. Comparison of endogenetic microbial community diversity between wild *Cordyceps sinensis*, artificial *C. sinensis* and habitat soil. *Chin. J. Chin. Mater. Medica* **2021**, *46*, 3106–3115.
84. Yao, Y.-S.; Zhu, J.-S. Indiscriminate use of the Latin name for natural *Cordyceps sinensis* and *Ophiocordyceps sinensis* fungi. *Chin. J. Chin. Mater. Med.* **2016**, *41*, 1316–1366.
85. Zhang, S.; Zhang, Y.-J. Molecular evolution of three protein-coding genes in the Chinese caterpillar fungus *Ophiocordyceps sinensis*. *Microbiol. China* **2015**, *42*, 1549−1560.
86. Zhang, S.; Zhang, Y.-J.; Liu, X.-Z.; Wen, H.-A.; Wang, M.; Liu, D.-S. Cloning and analysis of the *MAT1-2-1* gene from the traditional Chinese medicinal fungus *Ophiocordyceps sinensis*. *Fungal Biol.* **2011**, *115*, 708−714.
87. Zhang, S.; Zhang, Y.-J.; Shrestha, B.; Xu, J.-P.; Wang, C.-S.; Liu, X.-Z. *Ophiocordyceps sinensis* and *Cordyceps militaris*: Research advances, issues and perspectives. *Mycosystema* **2013**, *32*, 577−597.
88. Zhang, S.-W.; Cen, K.; Liu, Y.; Zhou, X.-W.; Wang, C.-S. Metatranscriptomics analysis of the fruiting caterpillar fungus collected from the Qinghai-Tibetan plateau. *Sci. Sinica Vitae* **2018**, *48*, 562−570.
89. Zhang, Y.-J.; Li, E.-W.; Wang, C.-S.; Li, Y.-L.; Liu, X.-Z. *Ophiocordyceps sinensis*, the flagship fungus of China: Terminology, life strategy and ecology. *Mycology* **2012**, *3*, 2–10. https://doi.org/10.1080/21501203.2011.654354.
90. Zhang, Y.-J.; Sun, B.-D.; Zhang, S.; Wàngmŭ; Liu, X.-Z.; Gong, W.-F. Mycobiotal investigation of natural *Ophiocordyceps sinensis* based on culture-dependent investigation. *Mycosistema* **2010**, *29*, 518–527.
91. Zhang, Y.-J.; Xu, L.-L.; Zhang, S.; Liu, X.-Z.; An, Z.-Q.; Wang, M.; Guo, Y.-L. Genetic diversity of *Ophiocordyceps sinensis*, a medicinal fungus endemic to the Tibetan Plateau: Implications for its evolution and conservation. *BMC Evol. Biol.* **2009**, *9*, 290. https://doi.org/10.1186/1471-2148-9-290.

92. Zhang, Y.-J.; Zhang, S.; Li, Y.-L.; Ma, S.-L.; Wang, C.-S.; Xiang, M.-C.; Liu, X.; An, Z.-Q.; Xu, J.-P.; Liu, X.-Z. Phylogeography and evolution of a fungal–insect association on the Tibetan Plateau. *Mol. Ecol.* **2014**, *23*, 5337–5355. https://doi.org/10.1111/mec.12940.
93. Zhao, Y.-N.; Zhang, J.-H.; Meng, Q.; Zhang, H.; Zhou, G.-L.; Li, M.-M.; Wu, P.-P.; Shu, R.-H.; Gao, X.-X.; Guo, L.; et al. Transcriptomic analysis of the orchestrated molecular mechanisms underlying fruiting body initiation in Chinese cordyceps. *Gene* **2020**, *763*, 145061. https://doi.org/10.1016/j.gene.2020.145061.
94. Zheng, P.; Wang, C.-S. Sexuality Control and Sex Evolution in Fungi. *Sci. Sin. Vitae* **2013**, *43*, 1090–1097.
95. Zhong, X.; Gu, L.; Wang, H.-Z.; Lian, D.-H.; Zheng, Y.-M.; Zhou, S.; Zhou, W.; Gu, J.; Zhang, G.; Liu, X. Profile of *Ophiocordyceps sinensis* transcriptome and differentially expressed genes in three different mycelia, sclerotium and fruiting body developmental stages. *Fungal Biol.* **2018**, *122*, 943–951. https://doi.org/10.1016/j.funbio.2018.05.011.
96. Zhu, J.-S.; Gao, L.; Li, X.-H.; Yao, Y.-S.; Zhou, Y.-J.; Zhao, J.-Q.; Zhou, Y.-J. Maturational alterations of oppositely orientated rDNA and differential proliferations of CG:AT-biased genotypes of *Cordyceps sinensis* fungi and *Paecilomyces hepiali* in natural *C. sinensis. Am. J. Biomed. Sci.* **2010**, *2*, 217–238. https://doi.org/10.5099/aj100300217.
97. Zhu, J.-S.; Guo, Y.-L.; Yao, Y.-S.; Zhou, Y.-J.; Lu, J.-H.; Qi, Y.; Chen, W.; Zheng, T.-Y.; Zhang, L.; Wu, Z.-M.; et al. Maturation of *Cordyceps sinensis* associates with co-existence of *Hirsutella sinensis* and *Paecilomyces hepiali* DNA and dynamic changes in fungal competitive proliferation predominance and chemical profiles. *J. Fungal Res.* **2007**, *5*, 214–224.
98. Zhu, J.-S.; Gray, G.M. Renaturative catalytic blotting of enzyme proteins. In *Protein Blotting: A practical Approach (IRL Series)*; Dunbar, B.S., Ed.; Oxford University Press: Oxford, UK, 1994; Chapter 17, pp. 221–238. https://doi.org/10.1093/oso/9780199634385.003.0017.
99. Zhu, J.-S.; Halpern, G.M.; Jones, K. The scientific rediscovery of an ancient Chinese herbal medicine: *Cordyceps sinensis*: Part II. *J. Altern. Complem. Med.* **1998**, *4*, 429–457. https://doi.org/10.1089/acm.1998.4.429.
100. Zhu, J.-S.; Li, C.-L.; Tan, N.-Z.; Berger, J.L.; Prolla, T.A. Combined use of whole-gene expression profiling technology and mouse lifespan test in anti-aging herbal product study. In Proceedings of the 2011 New TCM Products Innovation and Industrial Development Summit, Hangzhou, China, 27 November 2011; pp. 443–448. Available online: https://xueshu.baidu.com/usercenter/paper/show?paperid=08341c17fa58c8f85584b92572b90f75&site=xueshu_se (accessed on 30 Jannuary 2025).
